# Exploring community kangaroo mother care practices among NICU graduates: a prospective cohort study in South India

**DOI:** 10.1186/s12884-026-09492-5

**Published:** 2026-06-20

**Authors:** Sathya Jeganathan, Catherine Schwinger, Tor A. Strand, Ravishankar Veerasamy, Ingrid Kvestad

**Affiliations:** 1 Department of Pediatrics, Chengalpattu Medical College, Chengalpattu , 603111 India; 2 Centre for International Health, Department of Global Public Health and Primary Care, University of Bergen, PO Box 7804, Bergen , N-5020 Norway; 3 Department of Disease Burden, Norwegian Institute of Public Health, Bergen, Norway; 4 Department of Research, Innlandet Hospital Trust, Lillehammer, Norway; 5 Regional Centre for Child and Youth Mental Health and Child Welfare, NORCE, Norwegian Research Centre, Bergen, Norway; 6 Women’s clinic, Innlandet Hospital Trust, Lillehammer, Norway; 7 Gandhigram Institute of Rural Health, Dindigul, India

**Keywords:** Low birth weight infant, Community Kangaroo Mother Care (cKMC), NICU graduates

## Abstract

**Aim:**

To describe Kangaroo Mother Care (KMC) practice in the community (cKMC) two months after discharge from the Neonatal Intensive Care Unit (NICU). in South Indian mother-LBW infants.

**Method:**

A prospective study enrolling 420 dyads at discharge from the NICU with follow-up on cKMC practice two months after discharge. Factors associated with cKMC were explored using logistic regression.

**Result:**

Among the 420 enrolled, 2 (0.5%) infants died, and 12 (2.9%) were lost to follow-up. Of the remaining families, 25% (101) never practiced cKMC, effective practice was done by 19% (77). Infant birth weight ≥ 1.5 kg (OR: 3.1, 95% CI 1.8, 5.3) was associated with higher odds of practicing cKMC, while being born at term (OR: 0.5, 95% CI 0.3, 0.8) and mothers’ weight > 45 kg (OR: 0.3, 95% CI 0.1–0.7) was associated with lower odds of practicing cKMC. Continued KMC practice 48 h before discharge was associated with higher odds (OR: 3.4, 95% CI 1.8–6.2), while absence of father’s support was associated with lower odds (OR: 0.6, 95% CI 0.3, 1.0) of effective cKMC.

**Conclusion:**

The continuum of cKMC after discharge from the NICU was inadequate. Factors associated with cKMC practice should be considered when planning interventions to improve cKMC practices.

## Introduction

Over 20 million babies are born with low birth weight (LBW) each year [1]. The World Health Organization (WHO) has defined LBW as, “the weight at birth less than 2,500 grams which can be a consequence of preterm birth, being small for gestational age, or both” [1]. As per the National Family Health Survey data-5 from 2019 to 2021, the infant mortality rate in India is 35.2 per 1000 live births [2]. LBW contributes significantly to infant mortality, emphasizing the importance of care of LBW infants in health and social systems [3].

There are many strategies to improve the survival of LBW babies, such as providing standard thermal care with incubators, antenatal steroids and Continuous Positive Airway Pressure (CPAP) for respiratory distress [3]. WHO has recommended Kangaroo Mother Care (KMC) as a cost-effective, evidence-based, high impact intervention for the care of preterm and LBW new-borns at high risk of neonatal mortality and morbidity [3].

KMC was developed by Dr. Edgar Ray in 1978 with the aim to provide efficient thermal care for LBW infants [4]. KMC is a neonatal care practice which benefits all new-borns, particularly those born preterm, with LBW, or in need of immediate medical attention. In the Global position paper, WHO defines KMC practice as including the following key features: continuous and prolonged (8–24 h per day, for as many hours as possible) skin-to-skin contact, initiated immediately after birth, exclusive breastfeeding or breastmilk feeding when KMC is initiated in healthcare facilities, timely discharge either from the NICU to a lower level of care within the facility or discharge to home (with skin-to-skin contact and close monitoring) [5].

Thus, advantages of KMC go beyond thermal regulation, facilitating breastfeeding, infection control, and bonding, promoting the health and well-being of LBW new-borns. Most published literature about KMC practices comes from health facilities where care was taught and given under the supervision of skilled health workers [6–8]. There are studies about immediate KMC in the hospital and survival of LBW infants [9]. Formative research in India has shown the awareness of KMC benefits may increase the adoption rate at home. High adoption rate can be achieved through intense and skilled counselling with support [10].

Although there are studies about the benefits of KMC in reducing the mortality both in the hospital and community settings, little is known about the continuum of care of these LBW neonates in the transition from hospital care to the community [8, 11, 12]. There are studies in which there is mention about the duration of KMC, e.g. to assess effective KMC as more than 8 h per day [13, 14] and a recent study which mention the place of hospitalization as a determinant of practice of KMC after discharge [15]. Chengalpattu Medical College Hospital (CMCH) in the state of Tamil Nadu, South India offers KMC services to the LBW babies born in the advanced neonatal care centre, but the important question remains - how do families continue to practice KMC post discharge?

The objectives of the present study were to assess KMC practice at home (cKMC) among families with new-borns two months after discharge from the Neonatal Intensive Care Unit (NICU) at CMCH, and to identify factors associated with cKMC practice after discharge.

## Methods

### Study setting and population

The study participants were identified at CMCH, one of the largest perinatal care centres in the state of Tamil Nadu, South India with 10,000–12,000 deliveries per year. All mothers with high-risk pregnancies from Chengalpattu and neighbouring districts are referred to CMCH.

The CMCH has 66 bedded neonatal units, with 16 beds for intensive care and the rest for stepdown units. The health team provides guidance to KMC practice under the supervision of nurses, in addition, there are a KMC ward and a father Kangaroo ward adjacent to the NICU with comfortable reclining cots and chairs to facilitate family KMC practice. The hospital policy allows parents to stay 24/7 with their child in NICU as well as in designated stepdown wards. Through private public partnership, Ekam Foundation NGO was supporting the SNCUs in the government facilities by filling the gaps in terms of equipment support, manpower training and follow up services [16].

**Fig. 1:**
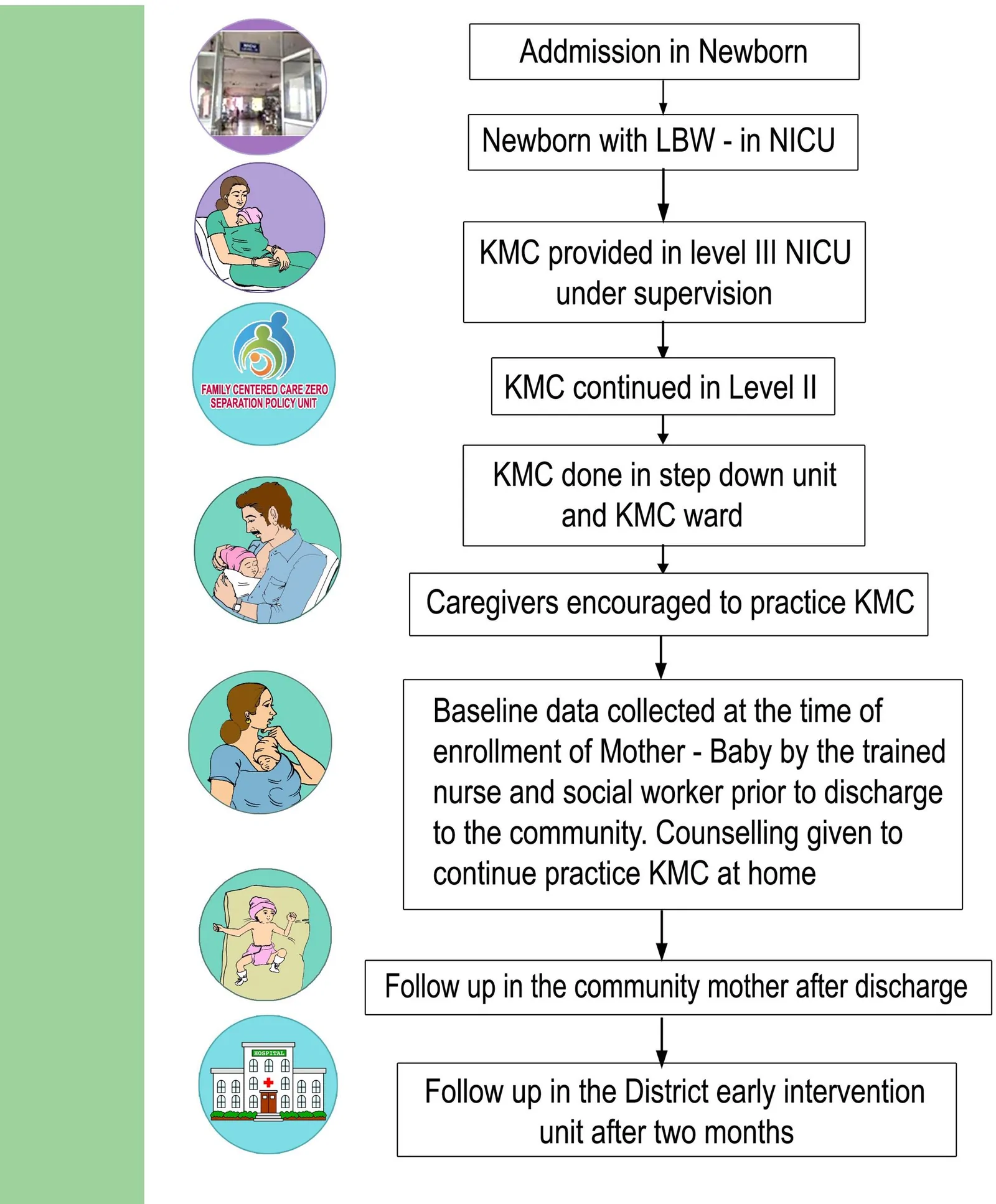
The continuum of kangaroo mother care practice for low birth weight infants in the health system in the state Tamil Nadu, South India

SNCU (sick New-born Care Unit) is a program to provide quality neonatal services in major government hospitals in Tamil Nadu [17]. It is a routine practice of the SNCU team (SNCU data analyst, follow up nurse, lactation counsellor) to counsel the families at the time of discharge regarding continuing skin-to-skin contact (SSC) and breast feeding. According to WHO guidelines, the usual practice at CMCH is to counsel the families to continue the KMC as long as possible, till the baby wriggles out the mother’s chest. Caretakers are encouraged to follow the practice at home. The continuum of KMC practice for LBW infants in the health system (both in the facility and the community) is depicted in Fig. 1.

### Study design and study sample

The study employed a prospective follow up design, enrolling families of LBW new-borns at discharge from the NICU, from January 2020 to December 2020. All neonates born alive at CMCH, admitted to NICU with a birthweight of less than 2,500 g were invited to participate and included after obtaining written consent from the mother/caregiver. LBW neonates delivered outside CMCH and referred from the community and those born from multiple pregnancies were also invited to the study. Neonates with the following criteria were excluded: (1) neonates born with life-threatening congenital malformations, (2) neonates of mothers with absolute contraindication to breastfeeding like carcinoma, (3) neonates abandoned by the family and discharged to child helpline services, (4) neonates currently participating in other clinical trials. The hospital has a general post-natal ward, but only neonates from NICU were enrolled.

There is a dearth of evidence in Tamil Nadu on community practice of KMC. Hence for the study, the proportion (P) is assumed to be 50%. Confidence interval taken as 95% (Z = 1.96) and Precision (d) is 5%. Applying these values in simple sample size formula, sample size arrived with 403 with non-response error, 5%. Mothers intending to stay away from the study area (18.5%) and who did not give consent (9.2%) were also excluded. Of a total 596 mothers were invited to participate in the study, 420 mother-baby dyads were enrolled (70.5%) [18].

### Data collection

Baseline data were collected at discharge from the NICU by a team of two trained nurses and one social worker and data entered using the mobile application Epicollect5, a data capture tool developed by the Centre for Genomic Pathogen Surveillance (CGPS) at the University of Oxford’s Big Data Institute (BDI). Socio-demographic details and mothers’ weight at first visit were obtained from the Pregnancy and Infant Cohort Monitoring and Evaluation (PICME) card and delivery details from hospital records. A pretested questionnaire, designed in a user-friendly manner, was used to collect information on the KMC practices in the hospital and at discharge. During pretesting, a small group of participants not included in the main study responded to the questionnaire. Their feedback was scrutinized and incorporated to refine the instrument. The team received comprehensive training in interviewing techniques and efficient data documentation methods before data collection, ensuring the accuracy and reliability of the gathered data.

The assessment was for Skin-to-Skin Contact practice (SSC) and breastfeeding by Ekam follow up nurse assisted by community district co-ordinator team. The end point of assessment of KMC practice was at two months post discharge regarding SSC (duration per day/week, position, family participation and exclusive breastfeeding or not).

Other studies as well as our pilot study, indicated KMC practices up to 2 months after discharge [4, 11]. To capture the full extent of KMC practices in our study while trying to minimize the recall bias, we decided to follow-up and assess LBW neonates at two months post-discharge, which is also a significant transition period for evaluating sustained breastfeeding practices, breastfeeding stability, and parental adaptation to KMC.

The cKMC practice was defined as any KMC practice in the community (yes/no). As per the operational guidelines of the Government of India on KMC for service care providers, the duration of cumulative completed hours of skin-to-skin contact in a 24-hour period in the following categories: short (less than 4 h daily), extended (5–8 h daily), long (9–12 h daily) and continuous (more than 12 h daily) [19]. As per this guideline, we collected duration of SSC practice as categories; short, extended, long and continuous. The study followed WHO guidelines, advising continued SSC as long as the mother and baby were comfortable till the baby wriggles out.

The questionnaire included questions on: cKMC practices: the frequency and duration of cKMC practices at home, as well as the position adopted by the mothers for KMC practice such as sitting, reclining and both positions. Facilities in home environment: essential amenities fostering KMC practice such as availability of a private room and a separate bed. Family Type: Nuclear family was defined as consisting of a married couple and their children living together in one household. A joint family was defined as including multiple generations living together in the same household. Father participation: whether other family members, such as fathers, participated in KMC (Kangaroo Father Care (KFC) is considered SSC practice as provided by the father). Challenges faced: questions concerning difficulties faced by the mothers [16]. Due to the ongoing COVID pandemic, when there was restriction of movement, data was collected by phone calls, tele monitoring and counselling done by follow up nurse. However in cases when there were difficulties encountered to contact, the district coordinator of Ekam with special permission visited the families at home to assess and support the families. Infant weight data during home visits were not collected due to COVID-19 restrictions.

### Statistical analysis

Following a thorough process of revision and validation, the data was exported to SPSS version 29 for subsequent analysis. The data set was subjected to a process of data cleaning that involved identifying the duplicate, incomplete entries, outliers and missing values. The data was checked for inconsistencies, reviewed, and rectified if possible.

The protocol from KMC Multicentric Study Group suggests that the recommended duration for practicing KMC is eight hours or more [13]. A systematic review and meta-analysis for KMC suggest that KMC should be provided for at least 8 h a day for greater benefit [20]. Hence, we defined effective KMC practice as at least 8 h per day with exclusive breastfeeding in the current study.

Descriptive statistics such as frequencies, proportions and means were calculated to summarize the characteristics of KMC practices. Logistic regression analysis was used to identify factors associated with practicing cKMC or not, and effective cKMC or not [21]. Families (*n* = 101) who did not practice cKMC at the follow-up were excluded from the analysis on effective cKMC or not.

For both outcomes, several potential independent variables were initially assessed, i.e., sex of the baby, gestational age (< 37 vs. ≥ 37 weeks), birth weight (≤ 1.5 vs. ≥ 1.5 kg), mode of delivery (normal vs. caesarean section), Parity (Primigravida and Second and more), delivery type (twin vs. singleton), mother’s age (< 25 vs. ≥ 25 years), religion (Hindu, Christian or Muslim), Education (illiterate, Primary Education, Secondary and above), mother’s weight (< 45 vs. ≥ 45 kg), monthly family income (< 8,000 INR, 8000-12,999 INR, ≥ 13,000 INR), Family type (nuclear vs. joint), initiation of KMC after birth in the hospital (before vs. after 3 days), KMC at discharge (effective/≥8 h vs. ineffective/<8 h) weight at discharge (≤ 2000 g and > 2000 g) and father support for KMC (yes vs. no). The statistical analysis utilized a stepwise approach to select variables, following the recommendations of Hosmer and Lemeshow [22]. Initially, the relationship between each independent variable and the chosen outcome (either cKMC practice or not, or effective cKMC or not) was evaluated using univariate logistic regression models. Variables that demonstrated statistical significance at a p-value of less than 0.2 were retained in multivariate logistic regression models, while those that did not show statistical significance in the initial assessment were reintroduced individually later into multiple models. Only variables that remained significant (*p* < 0.05) after this process were included in the final multiple models presented in the paper.

### Ethical considerations

This study acquired ethical committee approval from the Chengalpattu Medical College Institutional Ethics Committee and the Norwegian Regional Committee for Medical and Health Research Ethics (32413).

## Results

A total of 420 mother-baby dyads were enrolled at the time of discharge, and 406 of these were followed up in the community two months later. Two infants died before follow-up, and twelve families had moved out of the community and could not be contacted. Table 1 provides the baseline characteristics of the mother-baby dyads. Among the mothers, 25% (102) had term deliveries. The majority of the infants were first-born 73% (295) and delivered in the tertiary care medical college 94% (380). Of the infants, 97% were exposed to KMC in the hospital and only 3% (12) had not initiated KMC in the NICU and Stepdown ward.

**Table 1:** Baseline characteristics in a cohort of 406 low birthweight children discharged from the neonatal intensive care unit in South India

		*N*	%
Maternal and socio-economic characteristics			
Sex of the Baby	Female	186	46
	Male	220	54
Mothers age	≤ 21 Years	79	19
	22–30 Years	288	71
	> 30 Years	39	10
Mothers weight	Less than 45 Kg	56	14
	45Kg and above	350	86
Birth Weight	≤ 1500 g	145	36
	1501–2000 g	254	63
	≥ 2000 g	7	1
Religion	Hindu	402	99
	Muslim	1	0.2
	Christian	3	0.7
Educational status	Illiterate	22	5
	Primary	159	39
	Secondary and above	225	55
Family Type	Nuclear – Married couple and children in one household	112	28
	Joint Family- multiple generations living together in the same household	294	72
Family Income /Month	< Rs.8000	99	24
	Rs.8000- Rs.12,999	174	43
	≥Rs.13,000	133	33
Birth and new born characteristics			
Parity	Primigravida	225	55.4
	Second gravida and more	181	44.6
Gestational Age			
Extreme Preterm	< 28 weeks completed	2	0.4
Very Preterm	< 32 weeks	59	14.5
Moderate Preterm	32 to < 34 weeks	90	22.1
Late Preterm	34 to < 37weeks	153	37.7
Term	37 to 42 weeks	102	24.9
Twin birth		110	27.1
Place of birth	Medical College^1^	38 0	94
	Others^2^	26	6
Mode of delivery	Caesarean	197	49
New-born sex	Male	220	54.1
		Mean	SD
Gestational Age (Weeks)		34.4	2.5
Weight at Birth, g		1567	200
Weight at Discharge, g		2072	180

Mean ± Standard Deviation (SD) for continuous variables (Gestational Age, Weight at Birth, and Weight at Discharge)

^1^Tertiary care centre,^2^Referral from Primary Health Centre, District Hospital and Private Hospital

The cohort of 406 low birthweight neonates was predominantly male (54%), born to mothers aged 22–30 years (71%) via Caesarean section, and delivered at tertiary care medical college hospitals (94%). Late preterm births (34 to <37 weeks) constituted the largest gestational age category (37.7%), with a mean gestational age of 34.4 ± 2.5 weeks, and the majority were primigravida deliveries (55.4%). The mean birth weight and weight at discharge were 1,567 ± 200 g and 2,072 ± 180 g respectively, reflecting the low birthweight profile of the study population.

Table 2 presents information on cKMC practice two months after discharge. Approximately a quarter (101) of the families failed to engage in cKMC practice post-discharge, and 75% (305) adhered to cKMC practice. Only 19% (77) adhered effectively to the practices, and a mere 9% (35) followed the continuous practice of cKMC (≥ 12 h a day).

**Table 2:** Community Kangaroo Mother Care (cKMC) practice in 406 mother – low birth weights infants dyads two months after discharge from the neonatal intensive care unit in South India

Characteristics		*N*	%
Duration of Practice of cKMC after discharge (Months)	Not practiced	101	24.9
	More than one month	168	41.4
	Less than a month	137	33.7
Frequency of cKMC in a week	Not Practiced	101	24.9
	Daily	266	65.5
	4–5 times in a week	10	2.5
	Less than three times in a week	29	7.1
Frequency of cKMC in a day	Not Practiced	101	24.9
	Once	35	8.6
	Twice	77	19.0
	Thrice	88	21.7
	Four times and above	105	25.9
Hours of cKMC in a day	Not Practiced	101	24.9
	Short; <=4 h	134	33.0
	Extended; 5–8 h	94	23.2
	Long; 9–12 h	42	10.3
	Continuous; >=12 h	35	8.6
Home facilities for the 305 (75%) of families who practiced KMC			
a) Separate Bed availability at home	Yes	271	88.9
	No	34	11.1
b) Private Room	Yes	229	75.1
	No	76	24.9
c) KMC Position	Sitting in chair or floor	172	56.4
	In Sleeping position	61	20.0
	Both	72	23.6

Among those who practiced cKMC, 56% (172) usually opted for a sitting position, 20% (61) chose a reclined position, and 24% (72) practiced using both sitting and reclining positions. Regarding living arrangements, 75% (229) of households provided a private room and 89% (271) had a separate bed for the mother and baby.

Factors associated with practicing cKMC or not two months after discharge from NICU are shown in Table 3. In the multivariate model, the odds of practicing cKMC were lower among mothers with a weight of 45 kg and above (OR = 0.3 (95% CI 0.1, 0.7)) and in mothers who had delivered at term (OR = 0.4 (95% CI 0.3, 0.8)) compared to those who had delivered preterm. The odds of practicing cKMC were higher when the baby was born with a birth weight of 1.5 kg or above (OR = 3.1 (95% CI 1.8, 5.3)) and if the mother initiated KMC after 72 h of birth (OR = 2.8 (95% CI 1.4, 5.4)), compared to babies born with weight less than 1.5 kg and compared to initiating KMC earlier.

**Table 3:** Factors associated with practicing cKMC after discharge from NICU in 406 South Indian mother-low birth weight infant dyads

Characteristics		No cKMC practice after discharge *n* = 101	cKMC practice after discharge *n* = 305	Univariate logistic regression			Multivariable logistic regression§		
		*n* (%)	*n* (%)	OR	95%CI	*p*-value	OR	95%CI	*p*-value
Sex of the baby	Female	43(23.1%)	143(76.9%)	ref.					
	Male	58(26.4%)	162(73.6%)	0.8	0.5,1.3	0.451			
Gestational age	< 37 Months	60 (19.7%)	244 (80.3%)	ref.			ref.		
	≥ 37 Months	41 (40.2%)	61 (59.8%)	0.4	0.2, 0.6	< 0.001	0.5	0.3, 0.8	0.007
Birth weight	≤ 1.5Kg	48 (33.1%)	97 (66.9%)	ref.			ref.		
	> 1.5Kg	53 (20.3%)	208 (79.7%)	1.9	1.2, 3.1	0.005	3.1	1.8, 5.3	< 0.001
Mode of delivery	Normal	53 (25.4%)	156 (74.6%)	ref.					
	Caesarion	48 (24.4%)	149 (75.6%)	1.1	0.7, 1.7	0.817			
Parity	Prime	61(27.1%)	164(72.9%)	ref.					
	Second+	40(22.1%)	141(77.9%)	1.3	0.8,2.1	0.246			
Delivery type	Singleton	81 (27.4%)	215 (72.6%)	ref.					
	Multiple	20 (18.2%)	90 (81.8%)	1.7	1.0, 2.9	0.059			
Mothers age	< 25 Yrs	44(22.8%)	149(77.2%)	ref.					
	≥ 25 Yrs	57(26.7%)	156(73.3%)	0.9	0,7, 1.1	0.444			
Religion	Hindu	99 (24.6%)	303 (75.4)	1.2	0.3,4.6	0.807			
	Muslim	1 (100.0%)	0 (0%)	0.6	0.1,3.0	0.542			
	Christian	1 (33.3%)	2 (66.7%)	ref					
Educational status	Illiterate	4 (18.2%)	18 (81.8%)	ref					
	Primary	37 (23.3%)	122 (76.7%)	0.7	0.2, 2.3	0.594			
	Secondary and above	60 (26.7%)	165 (73.3%)	0.6	0.2, 1.9	0.39			
Mothers weight	< 45 kg	7 (12.5%)	49 (87.5%)	ref.					
	≥45 kg	94 (26.9%)	256 (73.1%)	0.4	0.2, 0.9	0.025	0.3	0.1, 0.7	0.008
Family income	< Rs.8000	19 (19.2%)	80 (80.8%)	1.2	0.6,2.2	0.627			
	Rs.8000–13,000	53 (30.5%)	121 (69.5%)	0.6	0.4,1.1	0.091			
	≥Rs.13,000	29 (21.8%)	104 (78.2%)	ref					
Family type	Nuclear	26 (23.2%)	86 (76.8%)	ref.					
	Joint	75 (25.5%)	219 (74.5%)	1.1	0.7,1.9	0.633			
Initiation of KMC after birth	< 3 days	88 (29.5%)	210(70.5%)	ref.			ref.		
	≥ 3 days	13 (12.0%)	95 (88.0%)	3.1	1.6, 5.8	0.001	2.8	1.4, 5.4	0.003
KMC at discharge	Ineffective(< 8 h)	87 (27.0%)	235 (73.0%)	ref.					
	Effective (≥ 8 h)	14 (16.7%)	70(83.3%)	1.9	1.0, 3.5	0.053			
Weight at discharge	≤ 2000 g	35(18.6%)	153(81.4%)	Ref.					
	> 2000 g	66(30.3%)	152(69.7%)	0.5	0.3,0.8	0.007			
Father support of KMC	Yes	0	109(100.0%)	ref					
	No	101(34.0%)	196(66.0%)	-	-	-			

§ r^=0.178 (17.8%)

Father’s support of cKMC was a determinant of cKMC practice after discharge. There was no support from the fathers among the mother-baby dyads that did not practice cKMC. Mode and type of delivery, mother’s age, educational status and family type at discharge were not associated with cKMC two months after discharge.

Factors associated with effective practice of cKMC are summarized in Table 4. Mothers who continued to practice KMC in the hospital for at least 48 h before discharge had higher odds of practicing effective cKMC at home (OR = 3.4 (95% CI,1.8,6.2)). Among the families without paternal support, the odds of practicing cKMC were lower compared to families with paternal support (OR = 0.6 (95% CI,0.3,1). None of the other factors assessed was significantly associated with effective cKMC.

**Table 4:** Factors for effective cKMC after discharge from NICU in 305 South Indian mother-infant dyads practicing cKMC

Characteristics		Ineffective<8Hrs(228)	Effective>=8Hrs (77)	Uni-variate logisticregression			Multivariate logistic regression*		
		n (%)	n (%)	OR	95%CI	p-value	OR	95%CI	p-value
Sex of the baby	Female	105(73.4 %)	38(26.6%)	ref.					
	Male	123(75.9 %)	39(24.1%)	0.9	0.5,1.5	0.616			
Gestational age	<37 Months	180(73.8%)	64(26.2%)	ref					
	≥37 Months	48(78.7%)	13(21.3%)	0.8	0.4, 1.5	0.43			
Birth weight	≤1.5 Kg	68(70.1%)	29(29.9%)	ref					
	>1.5Kg	160(76.9%)	48(23.1%)	0.7	0.4, 1.2	0.203			
Mode of delivery	Normal	118(75.6%)	38(24.4%)	ref					
	Caesarean	110(73.8%)	39(26.2%)	1.1	0.7, 1.8	0.715			
Delivery Type	Single	158(73.5%)	57(26.5%)	ref					
	Multiple birth	70(77.8%)	20(22.2%)	0.8	0.4, 1.4	0.432			
Parity	Prime	119(72.6%)	45(27.4%)						
	Second +	109(77.3%)	32(22.6%)	0.8	0.5,1.3	0.342			
Mothers age	<25Yrs	110(73.8%)	39(26.2%	ref					
	≥25 Yrs	118(75.6%)	38(24.4%)	1	0.7, 1.2	0.715			
Religion	Hindu	210(73.9%)	74 (26.1%)	0.9	0	0.999			
	Muslim	10 (76.9%)	3(23.1%)	0.9	0	0.999			
	Christian	8 (100.0%)	0 (0%)	Ref.					
Educational status	Illiterate	16(88.9%)	2(11.1%)	Ref.					
	Primary	90(73.8) %	32(26.2%)	3.2	0.7,14.7	0.232			
	Secondary and above	122(73.9%)	43(26.1%)	2.6	0.6,11.6	0.223			
Mothers weight	<45kg	30(61.2%)	19(38.8%)	ref					
	≥45 kg	198(77.3%)	58(22.7%)	0.5	0.2, 0.9	0.019			
Family income	< Rs.8000	57(71.3%)	23(28.8%)	0.7	0.4, 1.3	0.342			
	Rs. 8000-13000	95(78.5%)	26(21.5%)	0.9	0.5, 1.7	0.784			
	≥Rs. 13000	76(73.1%)	28(26.9%)	ref.					
Family type	Nuclear	66(76.7%)	20(23.3%)	ref					
	Joint	162(74.0%)	57(26.0%)	1.1	0.6, 2.1	0.616			
Initiation of KMC after birth	before 3 days	165(78.6%)	45(21.4%)	ref					
	after 3 days	63(66.3%)	32(33.7%)	1.9	1.1, 3.2	0.024			
KMC at discharge	Ineffective (<8 Hours)	37(52.9%)	33(47.1%)	ref					
	Effective (≥8 Hours)	191(81.3%)	44(18.7%)	3.9	2.2, 6.9	<0.001	3.4	1.8, 6.2	<0.001
Weight at discharge	≤2000g	114(74.5%)	39(25.5%)	ref.					
	>2000g	114(75%)	38(25%)	1.0	0.6,1.6	0.922			
Father support of KMC	Yes	75(68.8%)	34(31.2%)	ref			ref.		
	No	153(78.1%)	43(21.9%)	0.6	0.4, 1.1	0.076	0.6	0.3, 1.0	0.047

*r*^=0.128 (12.8%)

Moreover, our analysis revealed that the weight of the infant at birth, as well as the mother’s underweight, were factors linked to the practice of cKMC. Furthermore, the provision of support from the father, coupled with the hospital practice of continuation of KMC up-to 48 h prior to discharge, were associated with effective cKMC practice.

### Breastfeeding

Along with SSC, 82.8% of the mothers did exclusive breastfeeding with their infants till follow up. Among the mothers, 305 mothers (36%) who practiced cKMC felt that they gained lactational improvement due to SSC.

Supplemental Table 1 describes family characteristics and KMC. In the hospital 48 h prior to discharge, 97% (394) of the dyads practiced KMC, but only 21% (84) practiced effective KMC. Supplemental Table 2 reports barriers to cKMC practice. Maternal discomfort, particularly in back pain and post-caesarean pain, and heat-related discomfort is the main challenge in cKMC practice rather than fatigue or baby-related issues.

## Discussion

This study represents one of few studies conducted in South India delineating the practice of KMC for LBW new-borns discharged from a government NICU health facility. Among the larger states of India, Tamil Nadu (TN) has the second-lowest neonatal mortality rate [23], but to the best of our knowledge, there is no published literature from TN describing the continuum of KMC for new-borns discharged to the community.

Our findings underscore a significant inadequacy in the implementation of cKMC within the community setting. The KMC uptake study for small babies in Karnataka, India, has shown that almost three quarters of mothers continued KMC 30 ± 8 days after discharge [15]. A study in Ghana documented nearly ubiquitous home-based cKMC practice; one week post discharge, 99.5% were still practising KMC and a majority of them continued cKMC practice over four weeks of life [24]. It is noteworthy that the current study deviates from the observed prevalence trends, revealing that a substantial proportion of families refrained from cKMC post-discharge.

Among the difficulties expressed, body pain was reported by approximately 40% of the mothers. Similar findings were reported in other published studies, in which postpartum pain, depression and fatigue in the mothers can reduce the practice of KMC [25–27].

During the study period sling bags are not supplied by the facility, while Ekam NGO supported the supply to the government hospitals. The NICU staff counselled the mothers to use either the sling bag or use the saree or towels to support the baby. It will be ideal to include provision of sling bags to all KMC wards so that the mothers can walk around while practicing cKMC which may improve the adherence to practice, and body discomfort may be reduced.

A high-risk pregnancy is defined as “a pregnancy wherein the presence of any medical factor, either related to the mother or the foetus, has the potential to negatively influence the course of the pregnancy and its outcomes“ [28]. In the context of our study, we also considered familial and maternal characteristics. Factors such as parity, educational status, mother’s age, and family structure did not exhibit a significant impact on the adoption of cKMC, but maternal weight was associated with cKMC practice. Intriguingly, we observed a higher chance of cKMC practice among mothers with a weight below 45 kg as retrieved from the PICME card documented at the first visit to the health facility. It is important to note that mothers with lower body weights are more susceptible to adverse birth outcomes, such as preterm delivery, stillbirth, and LBW. A study from China regarding pre-pregnancy maternal weight and pregnancy outcomes found that underweight mothers delivered more LBW high-risk infants [29]. Essentially, this signifies the challenge of delivering a high-risk infant by mothers who themselves are at a higher risk due to their weight, a scenario where the mother’s inherent risk intersects with that of the new-born. Remarkably, we found that these high-risk mothers demonstrated a more successful continuation of cKMC practices at home. It is of significance to underscore that those mothers who delivered preterm were more prone to practicing cKMC.

From a public health perspective, early identification and targeted interventions for high-risk mothers are essential to improve neonatal outcomes. Antenatal counselling on KMC and breastfeeding should be integrated into maternal health programs to ensure these mothers are adequately prepared. Additionally, community health workers should receive training not only on identifying high-risk mothers, but also on managing high-risk births, emphasizing structured health education, postnatal follow-up, and support to enhance KMC adherence and improve infant survival rates. In the current study, families with VLBW infants (< 1.5 kg) practiced less cKMC hours in a day at home. The CMCH has a targeted intervention strategy in the NICU, having facilities for intensifying KMC exposure for families in both the intensive and step-down units, with infrastructure for KMC and Father KMC wards. The hospital has the policy to retain the neonates till they reach 1.8 Kg. Despite these efforts in the facility, the continuity of KMC practice was inadequate among LBW infants. The possible hypothesis could be that this could be due to the adequate weight gain and stabilisation before discharge. However, further research is needed to assess the duration of hospital practice, length of stay, rate of weight gain and post discharge practice of KMC.

The study examined how the initiation of KMC and the practice of KMC before discharge affected the continued practice of cKMC at home. A study in South India has shown that counselling, peer support and KMC case sheet improved facility based KMC, whereas KMC link card and communication for healthcare workers helped to sustain KMC at home [30]. A Cochrane review mentions that multi-country and community-based trials investigating the initiation of KMC at the health facility and its impact on neonatal survival, revealed that the time of KMC initiation in various studies ranged from 3 to 24 days after birth [20, 31]. A study conducted in Haryana, India, emphasized the initiation of KMC within the first 72 h of life and highlighted its positive influence on survival [32]. While there is ample literature on the immediate benefits of KMC and its impact on neonatal survival, there is a lack of information regarding how early initiation of KMC affects duration of KMC in the community. Further research is necessary to explore the effect of immediate KMC on cKMC practice, ensuring a seamless transition from healthcare facilities to the community setting.

The present study shows that families with paternal support practiced longer duration of cKMC daily. Similar critical roles by family members to enhance cKMC was documented in African and Scandinavian studies [33, 34], and similar findings were found in other studies where structured care educational programs for families at NICU have shown benefits [35]. Our results in conjunction with previous results, support the importance of availability of health facilities, and the policy of allowing family members in the NICU for KMC practice.

### Limitations

The main limitation of the present study is its focus on the regional context, which may limit the generalizability. In the purview of the COVID-19 pandemic at the time of data collection, the families had to change the usual pattern of family functioning during the lockdowns. Also, the Integrated Child Development Scheme centres were closed, and monitoring growth was difficult. Hence the continuum of care at the home may have been compromised due to the altered socioeconomic pattern and altered work pattern of health care workers during the pandemic. The post discharge weight gain, and the community health workers role in promoting KMC could not be documented. There is no previous study related to community KMC in the state of Tamil Nadu, India, hence the 50% proportion was taken for sample size calculation which could be a limitation. This study focused only on the mother-baby dyad discharged from the government NICU health facility. The status of babies delivered in private and other government care settings are not known.

## Conclusion

To conclude, the practice of KMC in the community was inadequate. Hospital-based interventions like initiation of KMC, effective practice up to discharge and training the spouse and other family members to assist in KMC could help to improve the continuum of KMC in the community. Further implementation and quality improvement studies are needed to strengthen the continuum of care of LBW infants.

## Data Availability

No datasets were generated or analysed during the current study.

## References

[1] Global nutrition targets 2025. low birth weight policy brie. [cited 2023 Oct 9]. Available from: https://www.who.int/publications-detail-redirect/WHO-NMH-NHD-14.5.

[2] National family health survey. 5 - Google Search. [cited 2023 Aug 7]. Available from: https://main.mohfw.gov.in/sites/default/files/NFHS-5_Phase-II_0.pdf.

[3] WHO recommendations for care. of the preterm or low-birth-weight infant. [cited 2023 Oct 9]. Available from: https://www.who.int/publications-detail-redirect/9789240058262. 36449655

[4] Boundy EO, Dastjerdi R, Spiegelman D, Fawzi WW, Missmer SA, Lieberman E, et al. Kangaroo Mother Care and Neonatal Outcomes: A Meta-analysis. Paediatrics. 2016;137(1):e20152238. [cited 2025 Mar 7]. https://www.ncbi.nlm.nih.gov/pmc/articles/PMC4702019/10.1542/peds.2015-2238 PMC4702019 26702029

[5] Kangaroo mother care: a transformative innovation in health care. Global position paper. [Internet]. [cited 2025 Feb 25].

[6] Parmar VR, Kumar A, Kaur R, Parmar S, Kaur D, Basu S, et al. Experience with Kangaroo mother care in a neonatal intensive care unit (NICU) in Chandigarh, India. Indian J Pediatr. 2009;76(1):25–8. 19390999 10.1007/s12098-009-0024-2

[7] Joshi M, Sahoo T, Thukral A, Joshi P, Sethi A, Agarwal R. Improving Duration of Kangaroo Mother Care in a Tertiary-care Neonatal Unit: A Quality Improvement Initiative. Indian Pediatr. 2018;55(9):744–7. 30345976

[8] Neogi SB, Chauhan M, Sharma J, Negandhi P, Sethy G, Ali SH. Rolling out of kangaroo mother care in secondary level facilities in Bihar-Some experiences. Indian J Public Health. 2016;60(4):302–8. 27976654 10.4103/0019-557X.195864

[9] WHO Immediate KMC Study Group, Arya S, Naburi H, Kawaza K, Newton S, Anyabolu CH, et al. Immediate “Kangaroo Mother Care” and Survival of Infants with Low Birth Weight. N Engl J Med. 2021;384(21):2028–38. 10.1056/NEJMoa2026486. PMC8108485 34038632

[10] Mazumder S, Upadhyay RP, Hill Z, Taneja S, Dube B, Kaur J, et al. Kangaroo mother care: using formative research to design an acceptable community intervention. BMC Public Health. 2018;18:307. [cited 2021 Dec 27]. https://www.ncbi.nlm.nih.gov/pmc/articles/PMC5833044 10.1186/s12889-018-5197-z PMC5833044 29499685

[11] Rasaily R, Ganguly KK, Roy M, Vani SN, Kharood N, Kulkarni R, et al. Community based kangaroo mother care for low-birth-weight babies: A pilot study. Indian J Med Res. 2017;145(1):51–7. [cited 2024 Jan 16]. https://www.ncbi.nlm.nih.gov/pmc/articles/PMC5460573 10.4103/ijmr.IJMR_603_15 PMC5460573 28574014

[12] Conde-Agudelo A, Díaz-Rossello JL. Kangaroo mother care to reduce morbidity and mortality in low birthweight infants. Cochrane Database Syst Rev. 2016;2016(8):CD002771. 10.1002/14651858.CD002771. PMC6464509 27552521

[13] Mony PK, Tadele H, Gobezayehu AG, Chan GJ, Kumar A, Mazumder S, et al. Scalingup Kangaroo Mother Care in Ethiopia and India: a multi-site implementation research study. BMJ Glob Health. 2021;6(9):e005905. 10.1136/bmjgh-2021-005905. PMC8438727 34518203

[14] Patawat M, Choudhary R, Jain MK, Chanchalani R, Jain A. Improving the Duration and Rate of Home-Based Kangaroo Mother Care: A Before-and-After Intervention Study. Cureus. 2023;15(4):e37861. [cited 2023 Jul 11]. https://www.ncbi.nlm.nih.gov/pmc/articles/PMC10204614/10.7759/cureus.37861 PMC10204614 37223204

[15] Washington M, Macaden L, Smith A, Selvam S, Mony PK. Determinants of Kangaroo Mother Care Uptake for Small Babies Along the Health Facility to Community Continuum in Karnataka, India. Glob Health Sci Pract. 2023;11(3):e2200457. [cited 2023 Oct 9]. https://www.ncbi.nlm.nih.gov/pmc/articles/PMC10285725/10.9745/GHSP-D-22-00457 PMC10285725 37348942

[16] EKAM FOUNDATION. [cited 2025 Mar 9]. Home. https://ekam.ngo/

[17] SNCU - Yahoo India Search Results. [cited 2025 Mar 9]. https://tncea.dmrhs.tn.gov.in/program/sncu.pdf

[18] Althubaiti A. Sample size determination: A practical guide for health researchers. J Gen Fam Med. 2022;24(2):72–8. 10.1002/jgf2. 36909790 PMC10000262

[19] National Health Mission - Google search. [cited 2023 Sep 9]. https://www.scribd.com/document/556365709/2014-Operational-Guidelines-KMC-Optimal-Feeding-of-Low-Birth-Weight-Infants

[20] Sivanandan S, Sankar MJ. Kangaroo mother care for preterm or low birth weight infants: a systematic review and meta-analysis. BMJ Glob Health. 2023;8(6): e010728. [cited 2023 Jul 12]. https://www.ncbi.nlm.nih.gov/pmc/articles/PMC10254798/10.1136/bmjgh-2022-010728 PMC10254798 37277198

[21] Hosmer DW, Lemeshow S, Sturdivant RX. Applied Logistic Regression. 1st ed. Wiley; 2013 [cited 2023 Oct 9]. (Wiley Series in Probability and Statistics). https://onlinelibrary.wiley.com/doi/book/10.1002/9781118548387

[22] Hosmer, et al. Applied Logistic Regression.pdf. 2013. [cited 2023 Oct 9]. https://mybiostats.files.wordpress.com/2015/03/model_building_strategies_and_methods_for_logistic_regression.pdf

[23] SRS_Bulletin_2020_Vol_55_No_1 - Google search [Internet]. [cited 2023 Oct 10]. https://censusindia.gov.in/nada/index.php/catalog/45567

[24] Nguah SB, Wobil PN, Obeng R, Yakubu A, Kerber KJ, Lawn JE, et al. Perception and practice of Kangaroo Mother Care after discharge from hospital in Kumasi, Ghana: A longitudinal study. BMC Pregnancy Childbirth. 2011;11:99. [cited 2025 Mar 8]. https://www.ncbi.nlm.nih.gov/pmc/articles/PMC3266193/10.1186/1471-2393-11-99 PMC3266193 22133462

[25] Chan GJ, Labar AS, Wall S, Atun R. Kangaroo mother care: a systematic review of barriers and enablers. Bull World Health Organ. 2016;94(2):130–J141. 26908962 10.2471/BLT.15.157818 PMC4750435

[26] Koreti M, Gharde PM. A Narrative Review of Kangaroo Mother Care (KMC) and Its Effects on and Benefits for Low Birth Weight (LBW) Babies. Cureus. 2022;14(11):e31948. 10.7759/cureus.31948. PMC9794926 36582577

[27] Seidman G, Unnikrishnan S, Kenny E, Myslinski S, Cairns-Smith S, Mulligan B, et al. Barriers and enablers of kangaroo mother care practice: a systematic review. PLoS ONE. 2015;10(5):e0125643. 25993306 10.1371/journal.pone.0125643 PMC4439040

[28] Leichtentritt RD, Blumenthal N, Elyassi A, Rotmensch S. High-risk pregnancy and hospitalization: the women’s voices. Health Soc Work. 2005;30(1):39–47. 10.1093/hsw/30.1.39. 15847236

[29] Wei J, Wang T, Shu J, Liu Y, Song X, Sun M, et al. Parental pre-pregnancy body mass index and risk of low birth weight in offspring: A prospective cohort study in central China. Front Public Health. 2022;10. [cited 2023 Oct 9]. https://www.frontiersin.org/articles/10.3389/fpubh.2022.1036689 10.3389/fpubh.2022.1036689 PMC9748483 36530688

[30] Jayanna K, Rao S, Kar A, Gowda PD, Thomas T, Swaroop N, et al. Accelerated scale-up of Kangaroo Mother Care: Evidence and experience from an implementation-research initiative in south India. Acta Paediatr Oslo Nor. 1992. 2023;112 Suppl 473:15–26. 10.1111/apa.16236 35146803

[31] Immediate “Kangaroo Mother Care” and Survival of Infants with Low Birth Weight. N Engl J Med. 2021;384(21):2028–38. [cited 2023 Aug 6]. 10.1056/NEJMoa2026486. PMC8108485 34038632

[32] Mazumder S, Taneja S, Dube B, Bhatia K, Ghosh R, Shekhar M, et al. Effect of community-initiated kangaroo mother care on survival of infants with low birthweight: a randomised controlled trial. Lancet Lond Engl. 2019;394(10210):1724–36. 10.1016/S0140-6736(19)32223-8. 31590989

[33] Leonard A, Mayers P. Parents’ lived experience of providing kangaroo care to their preterm infants. Health SA Gesondheid. 2008;13(4):16–28. [cited 2022 Jul 20]. https://hsag.co.za/index.php/hsag/article/view/401

[34] Blomqvist YT, Frölund L, Rubertsson C, Nyqvist KH. Provision of Kangaroo Mother Care: supportive factors and barriers perceived by parents. Scand J Caring Sci. 2013;27(2):345–53. [cited 2023 Oct 15]. https://onlinelibrary.wiley.com/doi/abs/10.1111/j.1471-6712.2012.01040.x 10.1111/j.1471-6712.2012.01040.x 22816503

[35] Samsudin S, Chui PL, Ahmad Kamar A, Abdullah KL, Yu CW, Mohamed Z. The Impact of Structured Kangaroo Care Education on Premature Infants’ Weight Gain, Breastfeeding and Length of Hospitalization in Malaysia. J Multidiscip Health. 2023;16:1023–35 10.2147/JMDH.S403206. PMC10106807 37077560

